# All Shades of Shrimp: Preferences of Colour Morphs of a Freshwater Shrimp *Neocaridina davidi* (Decapoda, Atyidae) for Substrata of Different Colouration

**DOI:** 10.3390/ani11041071

**Published:** 2021-04-09

**Authors:** Zuzanna Plichta, Jarosław Kobak, Rafał Maciaszek, Tomasz Kakareko

**Affiliations:** 1 Department of Ecology and Biogeography, Faculty of Biological and Veterinary Sciences, Nicolaus Copernicus University, Lwowska 1, 87-100 Toruń, Poland; z.r.plichta@gmail.com; 2 Department of Invertebrate Zoology and Parasitology, Faculty of Biological and Veterinary Sciences, Nicolaus Copernicus University, Lwowska 1, 87-100 Toruń, Poland; jkob73@umk.pl; 3 Department of Animal Genetics and Conservation, Institute of Animal Sciences, Warsaw University of Life Sciences, Ciszewskiego 8, 02-786 Warsaw, Poland; rafal_maciaszek@sggw.pl

**Keywords:** alien species, Eucaridea, Crustacea, polymorphism, ornamental species, aquarium trade, substratum selection, substratum colour, habitat preference, zoobenthos

## Abstract

**Simple Summary:**

Examination of preferences of an aquarium “Red Cherry” shrimp for differently coloured backgrounds revealed common traits, irrespective of shrimp body colouration. The shrimp selected dark backgrounds and coarse patterns over light and fine patterned substrata. Thus, the use of materials with dark and uniform colouration can contribute to designing proper monitoring tools to detect biological invasions after releasing this pet into the wild, as well as to provide shrimp with comfortable conditions in captivity.

**Abstract:**

An ornamental freshwater shrimp, *Neocaridina davidi*, is popular as an aquarium hobby and, therefore, a potentially invasive species. There is a growing need for proper management of this species to determine not only their optimum breeding conditions, but also their ability to colonise novel environments. We tested habitat preferences of colour morphs (brown, red, white) of *N. davidi* for substratum colour (black, white, grey shades, red) and fine or coarse chess-board patterns to recognise their suitable captivity conditions and predict their distribution after potential release into nature. We conducted laboratory choice experiments (*n* = 8) with three individuals of the same morph exposed for two hours to a range of backgrounds. Shrimp preferred dark backgrounds over light ones irrespective of their own colouration and its match with the background colour. Moreover, the brown and red morphs, in contrast to the white morph, preferred the coarse background pattern over the finer pattern. This suggests that the presence of dark, uniform substrata (e.g., rocks, macrophytes) will favour *N. davidi*. Nevertheless, the polymorphism of the species has little effect on its total niche breadth, and thus its invasive potential.

## 1. Introduction

Caridean shrimps have gained in popularity as an aquarium species due to their easy care, low maintenance costs, and multiple colour varieties obtained by selective breeding [1,2]. A freshwater Taiwanese shrimp, *Neocaridina davidi* (Bouvier, 1904), is one of the most common species for this hobby. Growing interest, leading to the release of shrimp by irresponsible aquarium owners, has resulted in establishing stable alien populations of *N. davidi* in Europe [2,3,4]. *N. davidi* lives in a wide variety of habitats: rivers, streams, and stagnant waters, both natural and anthropogenically modified [2,5,6]. They occur at shallow depths on submerged tree roots [2], macrophytes [3], or the leaf litter constituting part of their diet [7]. In colder climates, they often occupy artificially heated waters [3], but have also been reported thriving in a pond covered by ice in winter [2]. In the wild, shrimp can access habitats differing in colour, brightness, and texture. Thus, it is important for them to be able to match their colouration to the background, locate an optimum microhabitat, and protect themselves from predation by fish [8] and invertebrates [9].

Our purpose was to check the selectivity of *N. davidi* for differently coloured backgrounds and potential differences in substratum selectivity among its various colour morphs. The chances of particular morphs to become invasive may depend on the match between their colouration and substratum preferences. Knowledge of shrimp preferences would facilitate predictions of habitats susceptible to their invasion. It might also help breeders reduce the level of stress in shrimp, and therefore improve their well-being in captivity by the use of preferred backgrounds.

We chose three morphs with contrasting colourations: red, brown (corresponding to the wild type), and white. We hypothesised that: (1) Various morphs would differ either in their substratum colour preferences or in their match with the substratum colouration [10]; (2) The brown and red morph would show the highest preference for darker shades due to their dark colouration, whereas the white morph would choose lighter shades since its light colouration would be more visible on a dark background; (3) The brown morph would prefer a finely patterned background due to their spotted colouration, whereas more uniformly coloured red and white morphs would choose a coarse pattern.

## 2. Materials and Methods

We obtained captive bred shrimp from an ornamental shrimp farm (Kumak Shrimp, Konstancin–Jeziorna, Poland). They originated from clean variety lines in artificial pond cultures in Taiwan. We selected the following morphs (Figure S1): (1) red, with intense translucent red colouration, corresponding to the popular Red Cherry variety [11], (2) brown, with less intense translucent blueish colouration with black-brown spots, corresponding to the wild type occurring in native habitats, and (3) white, with translucent white colouration. In contrast to the other morphs used in the experiments, the white morph seems unable to change its colouration. This was obtained by artificial selection consisting of the elimination of all body pigments but the white one (personal information from the shrimp provider: R. Maciaszek). This has been confirmed in preliminary observations: these shrimp did not differentiate their colouration after a 3-month exposure to differently coloured backgrounds, as the other morphs normally do [10,11,12]. All shrimp used in the experiments had black pigmented eyes, indicating their non-impaired vision [10].

Shrimp were kept (each morph separately, 50 ind. per tank) in 84-L stock tanks (bottom: 60 × 40 cm, water level: 28 cm) equipped with filters, aerators, heaters, natural macrophytes and fine, light sand on the bottom. Thus, they could utilize substrata of the full range of shades, from the light bottom, to the dark filter surface. All of the tanks were situated in an air-conditioned room (20–21 °C) with a 12 h day: 12 h night photoperiod. The water parameters (measured with a multimeter Multi340i, WTW GmbH, Weilheim, Germany) were kept constant in the stock tanks and during the experiments (pH: 7.8, oxygen: 6.3 mg/L). Shrimp were fed every other day with “Mini Waffers mix” and “Shrimp Sticks” (Tropical, Chorzów, Poland). Food uneaten after a day was removed. Tank water (10–20%) was exchanged every three weeks.

The shrimp were measured from the tip of the rostrum to the posterior edge of the telson. The measurements were taken with ImageJ 1.52n software, [13] using video frames of the experimental recordings. Individuals from different morphs used in the experiments did not differ from one another in size (Kruskal–Wallis test: χ^2^ = 4.34, df = 2, *p* = 0.114). The mean length of shrimp used in the experiments was 16.4 mm (SD: 2.68 mm, range: 11.2–22.4 mm). We did not determine their sex, assuming that an animal has to be able to successfully hide from predators on the preferred background irrespective of its sex or life stage.

For the experiments, we used circular glass dishes (Figure S2) to eliminate shrimp accumulation in the corners. The backgrounds were prepared using Corel Draw X8 (Corel Corporation, Ottawa, Canada), printed out on printer paper, laid under the dishes, and wrapped around their sides (Figure S2). The whole setup was lit by two LED lamps (680 lux, 2800–3000K) and isolated by Styrofoam screens to prevent external disturbances. The locations of colour zones relative to the laboratory room were varied among replicates to exclude the effect of position on shrimp selection.

The experiments consisted of eight replicates (except Experiment 4, with *n* = 18). Each replicate involved three shrimp, placed into the experimental dish. Ca. 10 min before the experiment, shrimp were fed in the stock tank to standardize their hunger level. They were taken out of water for a few seconds to transport them to the experimental dish. Shrimp were given 1 h to adjust to the new environment, and then we recorded their behaviour (overhead video camera Samsung SNB-6004 with an objective lens 2.8-9 mm P-iris, SLA-M2890PN, Samsung, South Korea) for another 1 h.

We ran Experiment 1 on white, grey, and black background zones (Figure S2), preparing colours of greyscale values of 255, 128, and 0, respectively. As we observed shrimp preferences for the black background in this setup, (see the Results), we subsequently ran Experiment 2 to check their ability to discriminate between black colour (greyscale: 0) and two darker shades of grey (greyscales of 42 and 84). This allowed us to determine the finer-scale resolution of shrimp preferences. We also tested shrimp preferences for chessboard patterns (Experiment 3) with black (preferred) and white (avoided) squares (coarse pattern: 1-cm squares, fine pattern: 1-mm squares, Figure S2). Both patterns had the same mean greyscale values (equal areas of white and black colours). In Experiment 4, we tested the red morph on red (with red–green–blue components established as 255, 0 and 0, respectively) and grey (rgb: 85, 85, 85) backgrounds (with the same mean greyscale values, Figure S2) to check if they were sensitive to actual colours or shade differences.

We determined shrimp numbers occupying particular colour zones in video frames every 1 min. Then we calculated the occupation of colour zones in each replicate

$$Z=\frac{100\%*\sum_{i=1}^{t}n_{i}}{3t}$$

where: *Z*—occupation of the particular zone, *n_i_*—the number of shrimp in the particular colour zone in video frame *i*, *t*—number of the analysed video frames (*t* = 60), and 3—the number of shrimp in the container.

We tested shrimp preferences for particular zones using a one-sample Wilcoxon test, comparing the actual zone occupation with a theoretical value of 33.33% (for triple-choice tests, Experiments 1–2), or 50% (for pairwise choice tests, Experiments 3–4), assuming the random distribution of animals. We used Kruskal–Wallis tests (followed by pairwise Mann–Whitney U tests) to compare occupations of the same zones among the morphs.

We photographed three randomly selected shrimp of each morph on selected backgrounds using a Canon Powershot G10 (Tokyo, Japan) with manual mode on (aperture f/2.8, shutter speed 1/160, ISO speed 400 RGB colour space). The area was uniformly illuminated by the same light source as that used during the experiments. White balance was adjusted for colour correction by the use of a reference card. We used these photographs to measure the greyscale shades (from 0 for fully black colour to 255 for pure white) of backgrounds and shrimp bodies on various backgrounds with ImageJ. We determined modal greyscale values, i.e., dominating shades in the area. We conducted these measurements to determine the actual background colours and to check which backgrounds best matched the colourations of particular shrimp morphs. Actual background shades departed from the nominal values, but still differed from one another and showed a shade intensity gradient (Table 1). We also determined standard deviations (SD) of grey values of all pixels within the measured area of the shrimp body to determine the uniformity of their colouration. The SD values were measured separately for the entire shrimp body (excluding eyes) and for the abdomen only, to omit darker spots on the cephalothorax of the white and brown morphs (Figure S3). We split colour channels (red, green, blue) in ImageJ and measured individual components of shrimp colouration.

## 3. Results

**Figure 1:**
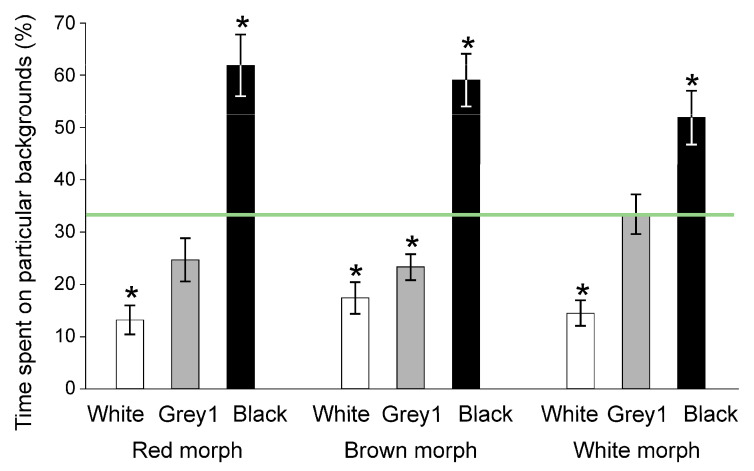
Preferences of shrimp morphs for differently coloured backgrounds in Experiment 1 (means ± SE). Asterisks show significant departures from the neutral selectivity (33.33%), indicated by the horizontal line. See Table 1 and Figure S2 for colour descriptions.

**Figure 2:**
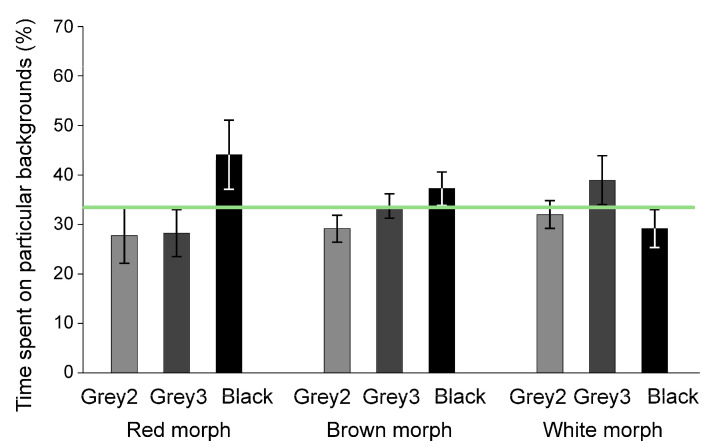
Preferences of shrimp for differently coloured backgrounds in Experiment 2 (means ± SE). Shrimp occurrences did not depart significantly from the neutral selectivity (33.33%, shown by the horizontal line) on any backgrounds. See Table 1 and Figure S2 for colour descriptions.

**Figure 3:**
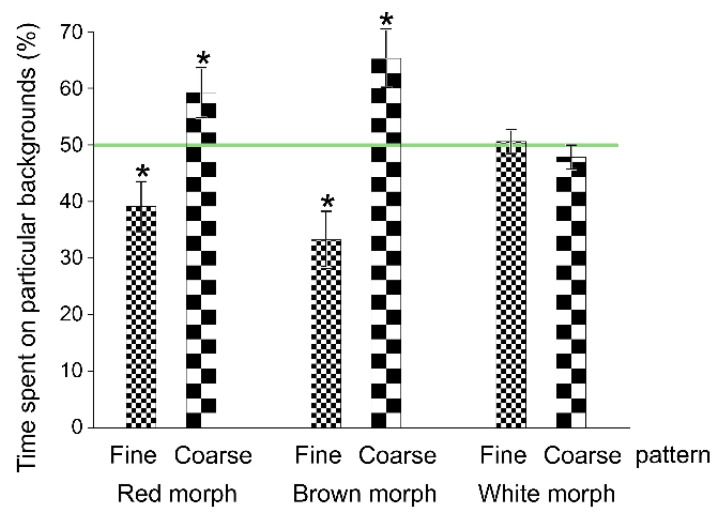
Preferences of shrimp morphs for different background patterns (fine and coarse black and white chessboard patterns with square sides of 1 and 10 mm, respectively) in Experiment 3 (means ± SE). Asterisks show significant departures from the neutral selectivity (50%) indicated by the horizontal line.

In Experiment 1, all the morphs selected the black background and avoided the white substratum (Figure 1, see Table S1 for the full statistics and Table S2 for the full raw data). In Experiment 2, all the morphs did not discriminate among the offered grey shades and the black background (Figure 2). In Experiment 3, the morphs differed in their preferences for background patterns: The red and brown morphs selected the coarse pattern, whereas the white morph did not discriminate between patterns (Figure 3). In Experiment 4, the red morph did not discriminate significantly (Table S1) between the red and matching grey backgrounds (56 vs. 44% of time, SE: 3.2%, respectively).

**Figure 4:**
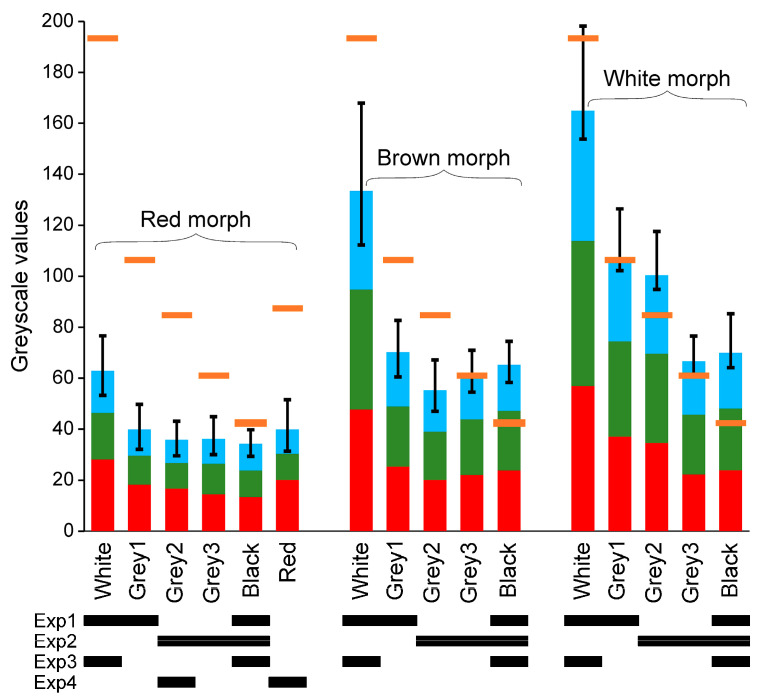
Measured (with ImageJ) shades (greyscale, 0: black, 255: white) of experimental backgrounds (orange bars) and shrimp bodies on the backgrounds (vertical bars). The modal values (the most common shades within the area) are shown. The bars are divided into red, green, blue components according to their shares in the body colouration. Error bars show the intraindividual standard deviations of the colour shades (the upper bar: the entire body, the lower bar: the abdomen only): the lower values indicate the more uniform body colouration. Black bars below the chart indicate experiments in which particular backgrounds and morphs were used.

The red, brown, and white morph shades were most similar to the black, darkest grey (grey3 in Table 1) and lightest grey (grey1 in Table 1) backgrounds, respectively (Figure 4). Shrimp colours were darker on darker substrata. The red morph was the darkest and the most uniformly coloured of the tested morphs, showing the lowest variation of body colouration among the backgrounds (Figure 4). The red component dominated in its colouration. The colourations of the other morphs were composed of similar shares of all three components. The colouration of the white morph was uneven due to dark spots on the cephalothorax and became uniform on the abdomen. The colouration of the brown morph was the most uneven due to dark spots on its entire body (Figure S1).

## 4. Discussion

As we expected, the brown (wild type) and red morph preferred darker shades, selecting the black background over grey, and avoiding the white bottom. However, contrary to our expectations, the white morph also selected the black background, indicating that all the morphs had similar shade preferences. We found no preferences of shrimp in Experiment 2, showing that they were able to discriminate between the grey shades of ca. 42 (the actual measured shade of the nominal black background, Table 1) and 106, but not between those of 42 and 85 (in the scale of 0–255). Thus, this seems to be the approximate threshold of shrimp capabilities of shade detection. The preference for darker habitats or shelters is a common phenomenon displayed by many organisms [14,15,16,17,18,19]. Dark substratum is often more similar to animal body colouration, allowing better camouflage, absorbing more heat, and providing better living conditions for heterotherms. On the other hand, light backgrounds may indicate the high access of light and a non-sheltered area [20]. Red shrimp did not discriminate between the red and grey backgrounds of the same grey shade, showing that their preference was based on shades rather than colours. In contrast, some crustaceans are able to select their backgrounds or shelters on the basis of true colour, rather than shade, though they also seem to avoid light objects [21].

The brown morph chose the coarse background pattern. Thus, they felt more hidden on bigger solid dark squares, even though their bodies could not completely fit into a single square. The finer pattern was not selected despite the spotted colouration of the wild morph. The relatively uniformly coloured red morph reacted similarly, preferring the coarse pattern, on which it was less conspicuous. In contrast to the other morphs, the white morph was indiscriminative with regard to the pattern size. This was the only case where the morphs varied among one another.

Our findings may be important for shrimp breeders. Using the optimum substratum colour may lead to their faster growth and lower aggression [16,22], and, as a consequence, to higher breeding success and more intense colouration [12,23].

The red morph was best camouflaged on the black background, which was also their preferred colour. The brown morph camouflage was best on the darkest grey background, which was also reflected in our results, since it did not discriminate between darker grey shades and the preferred black substratum. In accordance with their shade match, the white morph should have selected the grey background in Experiment 1. However, it also chose the black background, like the other morphs in our study. Thus, shrimp preferences do not match their actual colouration, but rather that of the wild morph, from which the other varieties originate. This may also mean that the white morphs have a lower chance of survival in the wild, since they chose suboptimal backgrounds.

Our results may be helpful to determine which habitats in a waterbody should be monitored to detect and prevent shrimp colonization at its early stage. The chances of finding *N. davidi* would be highest on dark uniform substrata (rocks, macrophytes) rather than on light sand of non-uniform colouration. Our findings may also be useful in designing artificial traps suitable for the monitoring and collection of animals in the wild [24]. Dark surfaces of homogenous colours seem suitable to attract animals and detect their presence.

Shrimp shades differed among substrata. Changes in shrimp colouration depend on physiological (chromatophore distribution) and morphological (pigment amount) changes in epidermal chromatophores [12,25]. The former mechanism is rapid and takes place within several minutes, whereas the latter takes days or weeks [12]. The physiological mechanism could contribute to the adjustment of shrimp to their backgrounds in our study. However, as the white morph is unable to actively control its body colouration, and yet looked different on various substrata, this was likely due to their translucency, meaning the background colour could be partially visible through their bodies.

Polymorphism shaped by natural selection increases the total niche breadth, distribution, and ability of species to deal with environmental changes [26,27]. In our study, the morphs showed different preferences for background pattern. This suggests that various morphs may select different habitat patches, which may enable them to occupy a wider range of microhabitats and increase the probability of survival in a novel area. Nevertheless, our study showed that the colour morphs did not differ in their shade preferences. Thus, there is little evidence that the polymorphism of *N. davidi* (obtained or enhanced through artificial selection) is associated with the enhanced habitat use and increases the likelihood of its success in the wild. Conversely, our study suggests that the morph colouration may compromise their survival in nature because of the impaired crypsis, particularly in the case of the white morph. It should be noted that natural colour polymorphism does not always result in different substratum preferences among the morphs [28]. Another potential benefit of polymorphism, potentially also applicable to shrimp, is the lower vulnerability to visual predators by the disruption of the prey search image [28].

## 5. Conclusions

We have demonstrated that the tested *N. davidi* morphs show a preference for the dark background, as well as avoidance of white colour. Furthermore, two out of the three tested morphs showed a preference for coarse background patterns compared to finer patterns. Our results can be useful for aquarium breeders as well as for predicting the future spread of the species into non-native habitats by using appropriate monitoring techniques.

## Figures and Tables

**Table 1:** Nominal (established in Corel Draw) and actual (modal values measured with ImageJ) greyscale shades (0 for full black, 255 for full white) of backgrounds used in the experiments.

Experiment 1	Greyscale Value		Experiment 2	Greyscale Value	
	Nominal	Measured		Nominal	Measured
White	255	193	Grey2	84	85
Grey1	127	106	Grey3	42	61
Black	0	42	Black	0	42
**Experiment 3**			**Experiment 4**		
White squares	255	193	Grey2	85	85
Black squares	0	42	Red	85	89

## Data Availability

The data presented in this study are available in Table S2.

## Supplementary Materials

The following are available online at https://www.mdpi.com/article/10.3390/ani11041071/s1, Figure S1: The red (a), brown (wild type) (b) and white (c) shrimp colour morphs used in the experiments. All individuals are adult females from the lines used in the study (photograph: Rafał Maciaszek), Figure S2: Experimental setup and backgrounds used in particular experiments (See Table 1 for actual measured greyscale values of the particular backgrounds), Figure S3: Areas within the shrimp body outline used to measure their colouration, Table S1: Statistical evaluation of shrimp preferences for particular backgrounds and inter-morph differences in background selection, Table S2. Raw experimental data.
