# Supplementary material for: All Shades of Shrimp: Preferences of Colour Morphs of a Freshwater Shrimp Neocaridina davidi (Decapoda, Atyidae) for Substrata of Different Colouration
Source: Animals (Basel). 2021 Apr 9;11(4):1071. doi: 10.3390/ani11041071 (PMC8069546; doi:10.3390/ani11041071)
Supplement: Supplementary file 1 [file animals-11-01071-s001.zip › animals-1125094- supplementary Figures S1-S3, Table S1 for conversion.pdf]

animals

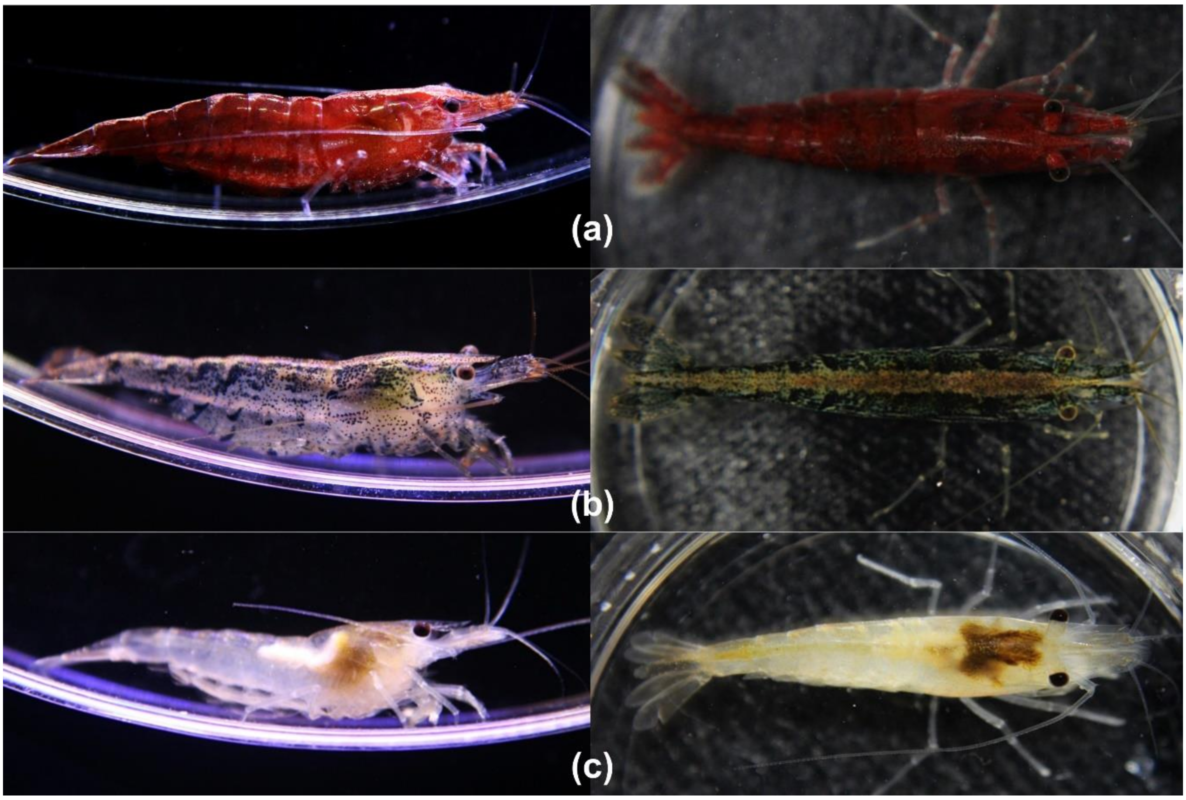

Figure S1. The red (a), brown (wild type) (b) and white (c) shrimp colour morphs used in the experiments. All individuals are adult females from the lines used in the study (photograph: Rafał Maciaszek).

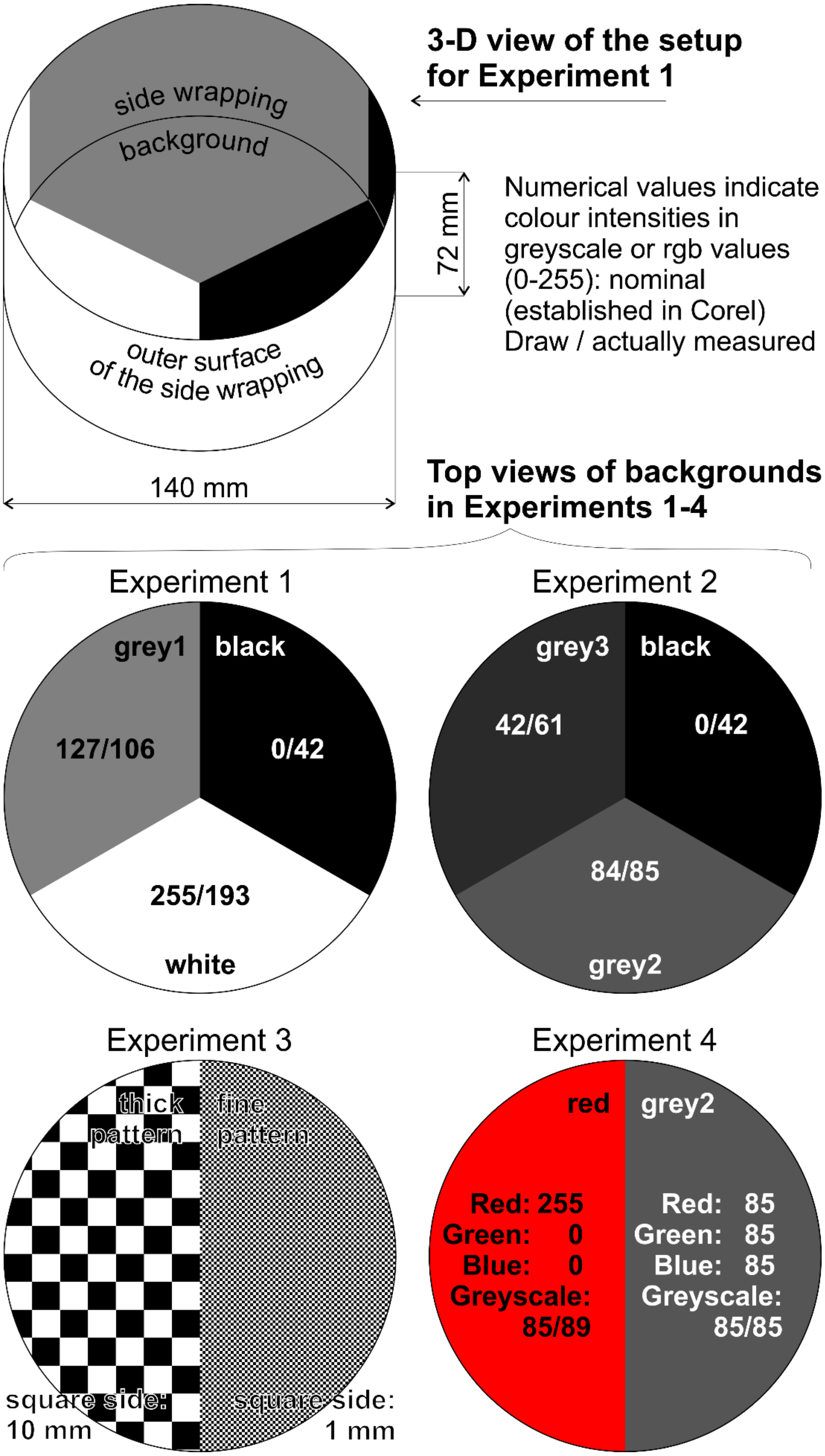

Figure S2. Experimental setup and backgrounds used in particular experiments.

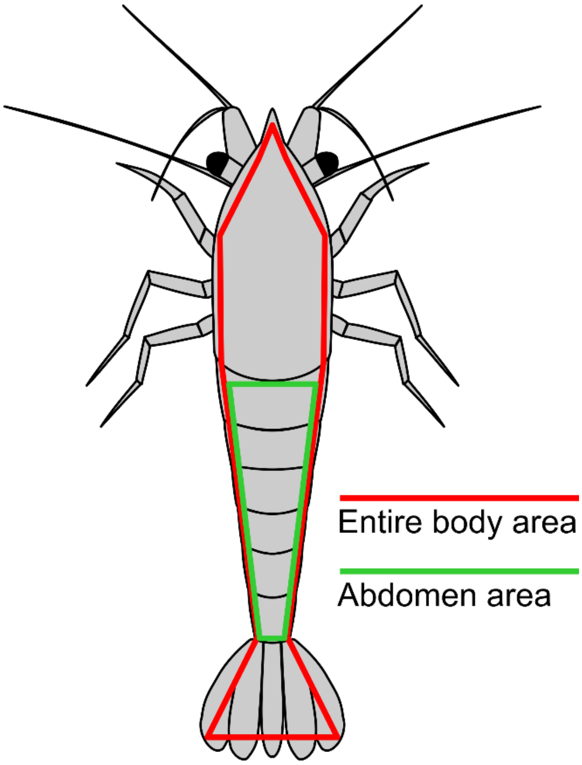

Figure S3. Areas within the shrimp body outline used to measure their colouration.

Table S1. Statistical evaluation of shrimp preferences for particular backgrounds and inter-morph differences in background selection. See Table 1 for background descriptions. Significant results are indicated with bold font.

Background	Differences Among Morphs (Kruskal-Wallis Tests)		Preferences of Morphs For Particular Backgrounds (Wilcoxon One-sample Tests)					
			Red morph		Brown Morph		White Morph	
	χ^2	*p*	z	*p*	z	*p*	z	*p*
Experiment 1								
White	1.41	0.493	-2.52	**0.012**	-2.52	**0.012**	-2.52	**0.012**
Grey1	5.93	0.052	-1.78	0.075	-2.37	**0.018**	-0.84	0.400
Black	2.57	0.277	-2.37	**0.018**	-2.52	**0.012**	-2.52	**0.012**
Experiment 2								
Grey2	0.50	0.777	-0.91	0.362	-1.12	0.263	0.00	1.000
Grey3	2.56	0.279	-1.19	0.233	-0.56	0.575	-0.68	0.499
Black	3.02	0.221	-1.54	0.123	-1.18	0.237	-0.63	0.528
Experiment 3								
Patterns	8.21	**0.017**	-1.96	**<0.050**	-2.24	**0.025**	-0.34	0.735
Differences among particular morphs in pattern preferences (Mann-Whitney U tests)			Red vs. Brown				-0.79	0.430
			Red vs. White				-2.00	**0.046**
			Brown vs. White				-2.73	**0.006**
Experiment 4								
Red vs. Grey2			-1.83	0.067				

Table S2. is provided separately, attached as an Excel File.
